# Lesion Topography Impact on Shoulder Abduction and Finger Extension Following Left and Right Hemispheric Stroke

**DOI:** 10.3389/fnhum.2020.00282

**Published:** 2020-07-17

**Authors:** Silvi Frenkel-Toledo, Shay Ofir-Geva, Nachum Soroker

**Affiliations:** ^1^Department of Physical Therapy, School of Health Sciences, Ariel University, Ariel, Israel; ^2^Department of Neurological Rehabilitation, Loewenstein Rehabilitation Hospital, Raanana, Israel; ^3^Sackler Faculty of Medicine, Tel Aviv University, Tel Aviv, Israel

**Keywords:** stroke, upper extremity, shoulder, abduction, finger, extension, brain mapping

## Abstract

The existence of shoulder abduction and finger extension movement capacity shortly after stroke onset is an important prognostic factor, indicating favorable functional outcomes for the hemiparetic upper limb (HUL). Here, we asked whether variation in lesion topography affects these two movements similarly or distinctly and whether lesion impact is similar or distinct for left and right hemisphere damage. Shoulder abduction and finger extension movements were examined in 77 chronic post-stroke patients using relevant items of the Fugl-Meyer test. Lesion effects were analyzed separately for left and right hemispheric damage patient groups, using voxel-based lesion-symptom mapping. In the left hemispheric damage group, shoulder abduction and finger extension were affected only by damage to the corticospinal tract in its passage through the corona radiata. In contrast, following the right hemispheric damage, these two movements were affected not only by corticospinal tract damage but also by damage to white matter association tracts, the putamen, and the insular cortex. In both groups, voxel clusters have been found where damage affected shoulder abduction and also finger extension, along with voxels where damage affected only one of the two movements. The capacity to execute shoulder abduction and finger extension movements following stroke is affected significantly by damage to shared and distinct voxels in the corticospinal tract in left-hemispheric damage patients and by damage to shared and distinct voxels in a larger array of cortical and subcortical regions in right hemispheric damage patients.

## Introduction

Stroke is a leading cause of adult acquired long-term motor disability (Langhorne et al., 2011). Up to 85% of post-stroke survivors present an initial upper limb (UL) motor deficit (Wade et al., 1983; Olsen, 1990), and up to 50% encounter UL function problems 4 years after stroke onset (Broeks et al., 1999). As a result, independence in activities of daily living, as well as the quality of life, remain reduced for most patients with severe hemiparesis (Urton et al., 2007). Upper limb rehabilitation trials designed to improve recovery rates have been largely unsuccessful (Krakauer and Carmichael, 2017), thus stressing the importance of obtaining a better understanding of the factors that limit and prevent the recovery process.

Recovery prediction for the hemiparetic upper limb (HUL) is important for setting a realistic rehabilitation goal, for planning a focused and personalized rehabilitation treatment program and for a more efficient allocation of resources. Early return of finger extension (FE; Fritz et al., 2005; Smania et al., 2007; Nijland et al., 2010; Stinear et al., 2012, 2017b; Winters et al., 2016; Snickars et al., 2017; Hoonhorst et al., 2018) and shoulder abduction (SA; Katrak et al., 1998; Nijland et al., 2010; Stinear et al., 2012, 2017b; Winters et al., 2016; Snickars et al., 2017; Hoonhorst et al., 2018) were found to be important prognostic determinants of subsequent HUL function after stroke. For example, in a cohort study, Nijland et al. (2010) found that stroke patients who exhibit some voluntary extension of the fingers and some abduction of the hemiplegic shoulder on day 2 have a 0.98 probability of regaining some dexterity at 6 months, whereas the probability was only 0.25 for those who did not exhibit this voluntary motor activity early after stroke onset. In another cohort study, Katrak et al. (1998) found that initial active shoulder abduction noted on average 11 days after stroke onset, predicted good hand movement at 1 month and hand function at 1 and 2 months. Bakker et al. (2019) even found that the ability of patients to voluntarily extend the fingers within 4 weeks after stroke was strongly related to Fugl-Meyer (FM) at 26 weeks after stroke, with no false-negative results and no additional value of the motor-evoked potential amplitude of the affected finger extension muscle for this clinical predictor. More recent studies have developed algorithms (also based on the scoring of SA and FE movements) to predict an individual’s potential for UL recovery within a few months post-stroke (Stinear et al., 2012, 2017b) and 2 years post-stroke (Smith et al., 2019). The implementation of the “Predict Recovery Potential” (PREP) algorithm for prediction of UL functionality in stroke rehabilitation, which combines clinical measures and neurophysiological and neuroimaging biomarkers, modified therapy content and increased rehabilitation efficiency after stroke, without compromising clinical outcomes (Stinear et al., 2017a).

Recovery of HUL function is constrained by the location of the anatomical damage (Grefkes and Fink, 2012; Grefkes and Ward, 2014; Frenkel-Toledo et al., 2019). During the acute phase, the probability of HUL recovery was found to decrease progressively with lesion location as follows: cortex—corona radiata—posterior limb of the internal capsule (PLIC; Shelton and Reding, 2001). In the sub-acute phase, damage to subcortical structures showed a higher association with poor motor performance of the HUL (Feys et al., 2000). We recently used voxel-based lesion-symptom mapping (VLSM; Bates et al., 2003) to investigate the impact of stroke lesion topography on HUL function (Frenkel-Toledo et al., 2019). Unlike various previous studies, where patients with right and left hemispheric damage (RHD, LHD) were grouped, the analysis of each patient group was done separately, given the differences between the dominant left and the non-dominant right cerebral hemispheres in the functional neuroanatomy of motor control (Tretriluxana et al., 2009; Mani et al., 2013), and known differences in patterns of motor recovery (Zemke et al., 2003; Wu et al., 2015). We found that in the sub-acute phase, HUL motor ability following LHD, assessed using the FM test, is affected mainly by damage to white matter tracts, the putamen, and the insula. In the chronic phase, FM performance in LHD patients was affected only by damage to white matter tracts. In contrast, HUL function following RHD was affected in both phases by damage to a large array of cortical and subcortical structures, notably the basal ganglia, white matter tracts, and the insula (Frenkel-Toledo et al., 2019).

Patients’ ability to execute voluntarily FE movement shortly after stroke onset is believed to depend on propagation of motor cortical impulses *via* the corticospinal tract (CST; Brodal, 2016; Stinear et al., 2017b), as the spinal motor neurons that innervate distal upper limb muscles are controlled almost exclusively by upper motor neurons of the lateral corticospinal system, which crossed from the contralateral side at the medullary level (Palmer and Ashby, 1992). By contrast, SA—a proximal UL movement, is likely to be controlled to some extent also by the anterior (ventral, non-crossing) CST and the descending brainstem tracts that maintain a more widespread bilateral innervation at the spinal level (Brodal, 2016). Despite this difference in innervation patterns, both early execution of FE and early execution of SA were found to constitute important prognostic determinants of HUL functional recovery, with quite similar sensitivity and specificity. For example, Snickars et al. (2017) found that a prognostic model based on FE at 3 days post-stroke onset plus stroke severity had sensitivity and specificity of 90.5% and 90.3%, respectively, and a model based on SA at 3 days and stroke severity had sensitivity and specificity of 85.7% and 82.3%, respectively.

The extremely high positive predictive value of early demonstration of SA and FE movement capacity (Katrak et al., 1998; Fritz et al., 2005; Smania et al., 2007; Nijland et al., 2010; Stinear et al., 2012, 2017b; Winters et al., 2016; Snickars et al., 2017; Hoonhorst et al., 2018) is likely to reflect the high correlation between the dynamics of SA and other shoulder-girdle movements, and high correlation of FE and other hand movements in the recovery process. This enables the use of these two movements as indicators of the capacity to move the proximal and distal segments of the HUL in activities of daily living. Unlike the high positive predictive value of SA and FE movements early after stroke onset (Nijland et al., 2010), the negative predictive value of these movements is much lower (i.e., lack of SA and FE movements early after onset does not preclude late recovery of HUL function). A substantial number of patients without initial voluntary FE movements experience a spontaneous return of these movements in the first 3 months post-stroke (Winters et al., 2016). According to the PREP-2 algorithm for prediction of HUL function at 3 months post-stroke, even in patients whose “SAFE” score at 3 days is poor (less than 5 of 10 Medical Research Council grades for SA plus FE), the combined use of the NIHSS score plus assessment of impulse propagation in the CST by transcranial magnetic stimulation (TMS), allows for the prediction of a poor, limited, or even good outcome (Stinear et al., 2017b).

The limited negative predictive power of SA and FE movement capacity early after stroke onset (Nijland et al., 2010; Stinear et al., 2012; Snickars et al., 2017) can be explained in different ways. One possibility is that HUL function recovers, despite the early lack of these movements, by resolution of reversible neurophysiological dysfunction in the motor network. Another option is that such recovery results from structure-function re-mapping processes and network re-organization, compensating for permanent focal structural damage in the motor system. It is unclear yet which of these explanations provides a better account of the limited negative predictive power in the current case. This information is important for guiding the development of interventions aimed to increase the HUL functional level in the chronic stage despite an early lack of SA and FE movement capacity. For example, it is crucial to know whether truncated transmission in the CST (evidenced by TMS) results from a reversible process or permanent structural damage to the tract, as in the latter case functional improvement may depend largely on the development of homolateral corticospinal control (Ward et al., 2006; Stinear et al., 2012, 2017b; Bradnam et al., 2013). Thus, in the case of CST permanent structural damage (but not in the case of reversible physiological impairment to CST connectivity), therapeutic interventions like non-invasive brain stimulation may need to target the intact hemisphere in an excitatory manner (Bradnam et al., 2012, 2013; Carmel et al., 2014; Harrington et al., 2020).

Discrimination between reversible and permanent damage to corticospinal control mechanisms is not easily obtainable from lesion analysis conducted at the time when SA and FE are examined for prognostication purposes, i.e., in the first few days after stroke onset. At this time the demarcation of the area of structural damage is not yet complete, especially in hemorrhagic stroke, and the final lesion boundaries may not be visualized clearly in CT/MR scans (Mikhael, 1989). The use of follow-up scans is more likely to provide the needed information concerning the functional neuroanatomy of SA and FE and the neuroanatomical constraints dictating their final level of recovery.

It should be noted that while the functional integrity of the CST originating from the primary motor cortex of the damaged hemisphere is believed to be necessary for the execution of distal upper-limb movements (hand and fingers, including FE) after stroke onset (Brodal, 2016; Stinear et al., 2017b; Bakker et al., 2019), the brain structures underlying the capacity to execute proximal movements, like SA, are less clear. Also, while the amplitude of motor-evoked potentials (MEPs) recorded from FE muscles early after stroke onset correlates with late FM scores, it was claimed that MEPs lack a significant added value for prediction of long-term hand function over and above the clinical prediction method (Bakker et al., 2019). This however might result from stimulation power being insufficient to activate functionally depressed but structurally preserved cortical motor neurons or reflect recovery processes based on structure-function re-mapping and network reorganization, involving brain systems that are not directly connected to the corticospinal pathways. Lesion studies may provide complementary information about the importance of specific brain structures, beyond the CST, for the execution of SA and FE movements after completion of the recovery process.

In the current study, we examined, for the first time, the long term impact of stroke lesion topography on patients’ capacity to execute SA and FE movements, beyond the time window where most neurological recovery occurs (Krakauer and Carmichael, 2017), that is—in the chronic phase of the disease, after completion of natural and treatment-related recovery, when patients’ capacity or incapacity to execute SA and FE movements stabilizes. At this stage, when focal structural brain damage is highly correlated with impaired task performance, the integrity of the damaged part of the brain is conceived to be necessary for the normal performance of the behavioral task in question. Following an earlier demonstration of dissimilar LHD and RHD lesion effects on motor function (Frenkel-Toledo et al., 2019) we assumed that hemispheric differences will be revealed for SA and FE and conducted VLSM analyses separately for LHD and RHD patient groups. We hypothesized that SA and FE are constrained mainly by damage to cortical and subcortical structures directly involved in motor execution, i.e., the upper-limb part of the homunculus in the primary motor cortex (pre-central gyrus) and the CST in its passage through the corona radiata and the PLIC (Brodal, 2016). Also, as both SA and FE are important prognostic determinants of overall HUL function, with quite similar sensitivity and specificity values (Snickars et al., 2017), we hypothesized that damage to common brain voxels and not only to different brain voxels will affect the ability to perform these two movements.

## Materials and Methods

### Participants

Seventy-seven first event stroke patients in the chronic stage (>1 year after onset) who were hospitalized in the subacute period at the Loewenstein Rehabilitation Hospital, Ra’anana, Israel, were recruited for the study. Patients were included if they did not suffer from previous psychiatric or neurological disorders, their language and cognitive status enabled comprehension of the task requirements and they did not have a subsequent stroke. The study was approved by the Ethics Review Board of the Loewenstein Hospital (approval number LOE-004-14). All participants were informed about the protocol and gave their written informed consent before inclusion in the study.

### Clinical Assessment

The standardized FM test (Fugl-Meyer et al., 1975; Gladstone et al., 2002) was used for the evaluation of HUL motor impairment. The test contains 33 test items for the HUL. These items are divided into four subsections: shoulder-arm, wrist, hand, and upper-limb coordination. Each test item is scored on a 3-point ordinal scale (0 = no movement, 1 = partial movement, 2 = full movement), with a maximal total score of 66 points. The scale has proven to be sensitive, reliable, and valid (Platz et al., 2005). For the current study, we performed lesion-symptom analysis on two items of interest from the FM test—finger extension and shoulder abduction (Nijland et al., 2010; Snickars et al., 2017; Hoonhorst et al., 2018). The chosen item for assessing shoulder abduction was “shoulder abduction 0–90°,” with starting position of the elbow at 0° and forearm in neutral position (and not the FM item that assesses abduction during flexor synergy), to assess a volitional movement with little or no synergy. Both finger extension and shoulder abduction were measured using the FM, similarly to the approach of Snickars et al. (2017).

### Imaging

Follow-up CT scans dated on average 51 and 28 days post-stroke onset for the LHD and RHD groups, respectively, were carefully examined by a physician experienced in the analysis of neuroimaging data (author NS). This was done to ensure that lesion boundaries were clear and traceable and that the CT presents a stable pattern of tissue damage without a mass effect from residual edema. Author NS was blinded to all other participants’ information.

### Lesion Analysis

Lesion analyses were performed with the Analysis of Brain Lesions (ABLe) module implemented in MEDx software (Medical-Numerics, Sterling, VA, USA). Lesion delineation was made manually on the digitized CTs. ABLe characterizes brain lesions in MRI and CT scans of the adult human brain by spatially normalizing the lesioned brain into Talairach space using the Montreal Neurological Institute (MNI) template. It reports tissue damage in the normalized brain using an interface to the Talairach Daemon (San Antonio, TX, USA; Lancaster et al., 2000), Automated Anatomical Labeling (AAL) atlas (Tzourio-Mazoyer et al., 2002; Solomon et al., 2007), Volume Occupancy Talairach Labels (VOTL) atlas (Lancaster et al., 2000; Solomon et al., 2007) or the White Matter Atlas (Mori et al., 2008). Quantification of the amount of tissue damage within each structure/region of the atlas was obtained as described earlier (Haramati et al., 2008). In the current study, tissue damage in the normalized brain was reported using the interface to the AAL and white matter atlases. Registration accuracy of the scans to the MNI template (Solomon et al., 2007) across all subjects ranged from 89.3% to 95.8% (94.2 ± 1.3%, 94.5 ± 0.8% in LHD and RHD subjects, respectively).

### Voxel-Based Lesion-Symptom Mapping (VLSM)

VLSM (Bates et al., 2003) was used to identify voxels (2 × 2 × 2 mm) of the normalized brain where damage has a significant impact on the SA and FE scores of the FM test. Voxel-by-voxel analysis was used to calculate the statistical significance of performance difference between subjects with and without damage in a given voxel, using the Mann-Whitney test. To avoid spurious results due to low numbers of lesioned voxels, only voxels lesioned in at least 10 subjects were tested (Rorden et al., 2007; Medina et al., 2010; Handelzalts et al., 2019) and at least 10 adjacent voxels had to show a statistically significant impact on performance for a cluster of voxels to be reported (McDonald et al., 2017). To correct for multiple comparisons, voxels with values exceeding a permutation threshold of *p* < 0.05 were considered significant (Mirman et al., 2018). Given the need to correct for multiple comparisons (as the basic anatomical unit in the analysis is a small volume of brain tissue, the voxel, of which there are hundreds of thousands in a brain), false-negative results are common in VLSM studies (Lorca-Puls et al., 2018). By setting a threshold of 10 subjects that had to have damage in a particular voxel for it to be included in the analyses, the number of comparisons that had to be corrected was reduced, thus increasing the statistical power of the analysis. Due to insufficient statistical power in one analysis, we also report anatomical regions containing clusters of at least 10 voxels, where patients affected in these voxels showed disadvantage relative to patients who were not affected in these voxels, using a lenient criterion of *p* < 0.01, which did not survive permutation correction for multiple comparisons (for a similar approach see references Schoch et al., 2006; Lo et al., 2010; Wu et al., 2015; Moon et al., 2016; Frenkel-Toledo et al., 2019). This information is provided under the assumption that in such cases VLSM points to possible trends. The maximum z-score is reported for each cluster of contiguous above-threshold voxels. Since, there may be multiple voxels with this maximum z-score in the cluster, we report the coordinates of the voxel that is most superior, posterior and left in its location within the cluster (the centroid of the cluster is not reported as it may not have the highest z-score value and it may not be an above-threshold voxel). The AAL atlas for gray matter and the White Matter Atlas (Lancaster et al., 2000; Tzourio-Mazoyer et al., 2002; Solomon et al., 2007; Mori et al., 2008) were used to identify the brain structures in which the significant clusters are located. Conjunction analysis was used to characterize voxels surpassing the VLSM thresholds (corresponding to z scores used for determining results that passed permutation correction for multiple comparisons or, in the case of FE in the LHD group, results that did not survive the permutation correction but passed the more lenient criterion of z score = 2.00) involved non-specifically in both the SA and FE and specifically in either the SA (*z* = 3.26 and *z* = 2.91 in LHD and RHD patient groups, respectively) or FE (*z* = 2.00 and *z* = 2.76 in LHD and RHD patient groups, respectively) by overlaying significant voxels from each analysis on the same template.

To rule out the possibility that the results were influenced differently in the RHD and LHD groups by demographic and clinical characteristics, gender, age, dominance, lesion type, time after stroke onset, lesion volume, SA and FE scores of the FM test, FM total score (FM T), Box and Blocks (B&B) and the FM sensation score were compared between groups, using *t*-tests or Mann-Whitney tests or Chi-square tests as required (normal group distribution of continuous data was assessed using Kolmogorov–Smirnov tests). A comparison was made also between the RHD and LHD groups concerning: (1) the proportion of subjects affected in each region of the AAL and WM atlases; and (2) the extent of damage in each region, using Chi-square/Fisher’s exact tests, and Mann Whitney tests, respectively. Also, correlations between total lesion volume and SA or FE scores were calculated in both groups, using Spearman-rho. False Discovery Rate (FDR; Genovese et al., 2002) was used to correct for multiple comparisons. All the tests were done using SPSS (version 25.0) with significance levels of *p*_FDR_ < 0.05.

## Results

As can be seen in Table 1, the demographic and clinical characteristics of the LHD and RHD patient groups are essentially similar. Individual data are displayed in the Supplementary Table S1.

**Table 1:** Demographic and clinical characteristics of participants.

	LHD group (*n* = 44)	RHD group (*n* = 33)	*p*-value
Gender (M/F)	32/12	25/8	0.764^a^
Age: Mean ± SD (range)	59.7 ± 10.5 (27.3–78.8)	63.2 ± 9.6 (26.7–73.2)	0.137^b^
Dominance (R/L/A)	41/2/1	30/2/1	0.934^a^
Lesion type (I/H/I>H)	2/41/1	2/30/1	0.633^a^
TAO (months): Mean ± SD (range)	27.5 ± 13.7 (12.0–59.8)	29.7 ± 13.9 (13.1–68.5)	0.510^c^
Lesion volume (cc): Mean ± SD (range)	26.4 ± 34.3 (0.4–182.3)	31.6 ± 41.8 (0.3–186.9)	0.853^c^
SA (0/1/2), Median (IQR)	12/8/24 2 (0–2)	11/1/21 2 (0–2)	0.743^c^
FE (0/1/2), Median (IQR)	8/9/27 2 (1–2)	9/5/19 2 (0–2)	0.573^c^
FM Total (X/66): Mean ± SD (range)	45.0 ± 18.6 (3–66)	43.3 ± 24.4 (4–66)	0.654^c^
B&B*: Mean ± SD (range)	29.9 ± 24.0 (0–67)	27.6 ± 23.0 (0–62)	0.585^c^
FM Sensation (X/12): Mean ± SD (range)	8.7 ± 4.2 (0–12)	8.6 ± 3.6 (2–12)	0.652^c^

LHD, left hemisphere damage; RHD, right hemisphere damage; Gender, M, Male; F, Female; Dominance R, Right; L, Left; A, Ambidextrous; Lesion type I, Ischemic; H, Hemorrhagic; I>H, Ischemic with hemorrhagic transformation; TAO, Time after stroke onset—mean (SD); SA, Shoulder abduction; FE, Finger extension; IQR, interquartile range; FM, Fugl-Meyer; B&B, Box and Blocks; *Number of participants in the B&B test: *n* = 30 (right) and *n* = 35 (left); Number of participants in the FM sensation test: *n* = 30 (right) and *n* = 39 (left); ^a^Chi-Square test, ^b^t-test, ^c^Mann Whitney test.

**Figure 1:**
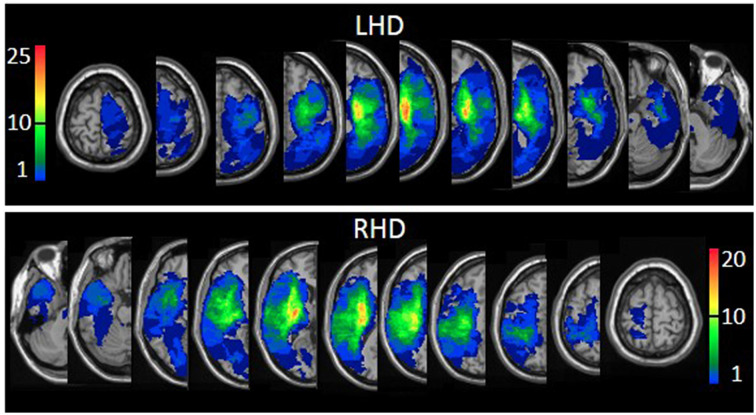
Lesion overlay maps of left hemispheric damage (LHD; *n* = 44) and right hemispheric damage (RHD; *n* = 33) patient groups. The threshold for inclusion in the Voxel-based Lesion-Symptom Mapping (VLSM) analysis: at least 10 subjects had to have damage to a particular voxel for it to be included in the analysis. Representative normalized slices (out of 90 normalized slices employed) are displayed in radiological convention (right hemisphere on the left side and vice versa), with warmer colors indicating greater lesion overlap (units: number of patients with a lesion in this region).

The proportion of LHD and RHD subjects having a lesion (yes/no) in at least 1 voxel in each of the regions of the AAL (Tzourio-Mazoyer et al., 2002; Solomon et al., 2007), and White Matter (Mori et al., 2008) atlases was similar, except for a larger proportion of LHD patients with damage to the caudate nucleus (73% vs. 46%, *p* = 0.019; FDR corrected *p* = 1.00). The extent of damage in each region was similar in LHD and RHD groups. In the RHD group, total hemispheric volume loss correlated significantly with SA (Spearman-rho = −0.570; FDR corrected *p* = 0.002) and FE (Spearman-rho = −0.516; FDR corrected *p* = 0.002). In the LHD group, total hemispheric volume loss did not correlate with SA or FE. Overlay lesion maps (stroke lesion distribution) of LHD and RHD patients are shown in Figure 1. Individual lesion data are displayed in Supplementary Figure S1.

**Figure 2:**
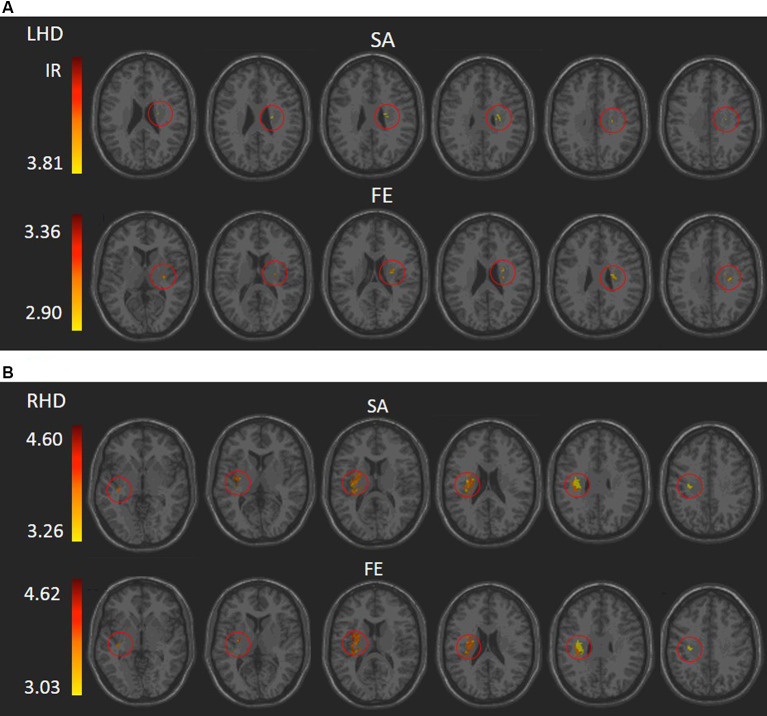
VLSM analysis depicting areas where damage was significantly associated with a lower score of shoulder abduction (SA) and finger extension (FE) in the LHD **(A)** and RHD **(B)** groups, respectively (minimum cluster size: 10 voxels, minimum number of patients affected in a voxel: 10). Warmer colors indicate higher z-scores. The colored regions in SA and FE of the RHD group and SA of the LHD group survived permutation correction for multiple comparisons, and the colored regions in the FE of the LHD group did not but were based on a lenient criterion of z score ≥ 2.00 (*p* ≤ 0.01). IR, irrelevant, all structures shared a single z score.

VLSM (Bates et al., 2003) identified clusters of voxels associated with poorer ability to perform SA and FE movements (Figure 2). Tables s 2, 3 show the anatomical structures in the left and right hemispheres, respectively, where damage was found to exert a significant impact on the tested abilities. In the LHD group (Table 2), both SA and FE movements were affected only by damage to the CST in its passage through the superior part of the corona radiata (SCR). The VLSM result for FE did not survive the correction for multiple comparisons by permutation. In the RHD group (Table 3), the lesion effect differs from the pattern observed in the LHD group. Here SA and FE movements were affected mainly by CST damage within the PLIC and SCR, as well as by damage to the external capsule (EC), association fibers of the superior longitudinal fasciculus (SLF), the insular cortex and the putamen.

**Table 2:** VLSM results in LHD patients (*n* = 44).

Test	Structure	*Z*-value	*X*	*Y*	*Z*	Voxels	% area
SA*	SCR	3.81	−24	−8	30	19	2.06
FE	SCR	3.36	−24	−12	26	59	6.39
		2.90	−28	−10	20	20	2.16

*SA results passed permutation correction for multiple comparisons (corresponding in this analysis to z scores of 3.269). FE did not survive the permutation correction but passed the more lenient criterion of z score = 2.00 or above. SCR, superior corona radiate.

**Table 3:** VLSM results in RHD patients (*n* = 33).

Test	Structure	*Z*-value	*X*	*Y*	*Z*	Voxels	% area
SA*	SCR	4.60	30	−16	30	171	18.59
	SLF	4.60	32	−16	30	151	18.30
	Insula	4.15	34	−12	16	143	8.08
	EC	3.83	32	−10	12	97	20.82
	Putamen	3.68	30	−10	6	71	6.67
		3.26	30	12	10	13	1.22
	PLIC	3.53	28	−14	18	40	7.98
	PCR	4.24	28	−22	26	26	5.75
	RLIC	3.68	28	−24	14	20	6.33
FE*	SCR	4.62	30	−14	30	174	18.91
	SLF	4.30	34	−12	30	142	17.21
	EC	3.39	30	−8	12	85	18.24
	Insula	3.67	34	−12	16	84	4.75
	Putamen	3.64	30	−10	6	75	7.05
		3.03	30	12	10	13	1.22
	PLIC	3.39	26	−8	14	60	11.98
	PCR	3.97	28	−22	26	24	5.31
	RLIC	3.64	28	−24	14	20	6.33
	ALIC	3.39	24	−4	18	11	2.70

*SA and FE results passed permutation correction for multiple comparisons (corresponding in these analyses to z scores of 2.917 and 2.763, respectively). EC, external capsule; SCR/PCR, superior/posterior corona radiata; PLIC/RLIC/ALIC, posterior/retro-lenticular/anterior limb of internal capsule; SLF, superior longitudinal fasciculus.

**Figure 3:**
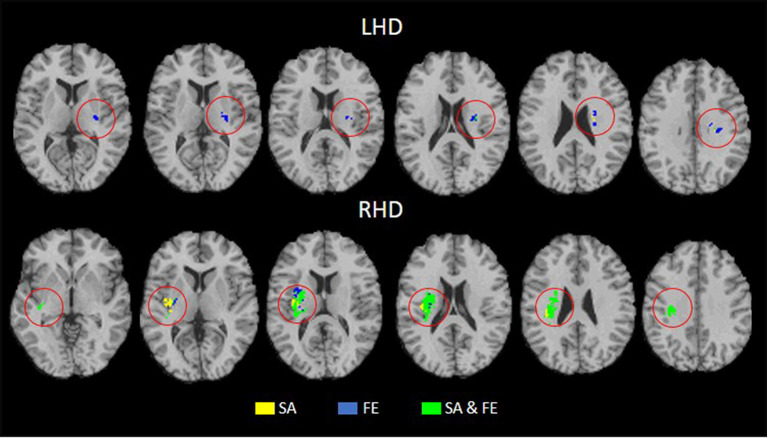
Conjunction analysis depicting areas of brain damage that were associated with lower scores in the SA only, FE only, and both SA and FE, shown in yellow, blue, and green, respectively, in the LHD (*n* = 44) and RHD (*n* = 33) groups.

Tables s 4, 5 show the anatomical structures in the left and right hemispheres, respectively, where VLSM conjunction analysis disclosed voxel clusters in which damage exerts a significant impact on SA only, FE only, and both SA and FE. The anatomical structures involved are shown in Figure 3. In the LHD group (Table 4), 50% of the voxels in which damage was shown to affect SA were involved selectively in SA, and 50% were involved both in SA and FE. In contrast, in 93% of the voxels in which damage affected FE performance, the impact of damage was specific to FE, while damage to only 7% of the voxels affected both SA and FE. As can be seen in Table 4, SA and FE movements were affected by lesions to common and distinct voxels of the SCR. In the RHD group (Table 5) the picture is different. In the large majority of voxels in which stroke lesion was shown to affect either SA or FE movements, the impact of structural damage was significant both to SA and FE. Moreover, in contrast to the findings of the LHD analysis where damage affecting both SA and FE was restricted to the CST in its passage through the corona radiata, in the RHD group, brain voxels in which damage affected both SA and FE were found in a large array of structures (Table 5). Selective impact on SA only was shown in 15% of the voxels where damage affected SA performance, and selective impact on FE only was shown in 9% of the voxels where damage affected FE performance. Thus, in the RHD group, the impact of damage to the large majority of “significant” voxels for either SA or FE was not specific and was noted in both SA and FE.

**Table 4:** Number of voxels in affected brain regions where the damage had a significant impact on SA only, FE only, and SA plus FE, in LHD patients (*n* = 44).

Areas	SA only	FE only	SA plus FE
SCR	6	63	6
Putamen		12	
EC		11	

*Only structures with at least 10 voxels affecting performance in one or more of the three options are shown. SCR, superior corona radiata; EC, external capsule*.

**Table 5:** Number of voxels in affected brain regions where the damage had a significant impact on SA only, FE only, and SA plus FE, in RHD patients (*n* = 33).

Areas	SA only	FE only	SA plus FE
SLF	13	4	141
Putamen	8	13	76
EC	32	21	74
SCR	1	4	177
Insula	59	2	84
PLIC		20	40
RLIC			20
PCR	2		24

*Only structures with at least 10 voxels affecting performance in one or more of the three options are shown. SLF, superior longitudinal fasciculus; EC, external capsule; SCR/PCR, superior/posterior corona radiata; PLIC/RLIC, posterior/retro-lenticular limb of internal capsule*.

## Discussion

The current study aimed to assess the impact of lesion topography on the capacity of chronic stroke patients to execute SA and FE movements in the HUL. The interest in these two specific movements stems from the fact that they seem to represent successfully the recovery process of proximal and distal muscle groups, thus pointing to the functional level of the HUL as a whole. This is evidenced by the fact that a patient who demonstrates SA and FE movement capacity a few days after stroke onset has an extremely high likelihood to manifest good HUL functionality in the chronic stage (Katrak et al., 1998; Fritz et al., 2005; Smania et al., 2007; Nijland et al., 2010; Stinear et al., 2012, 2017b; Winters et al., 2016; Snickars et al., 2017; Hoonhorst et al., 2018).

We used VLSM (Bates et al., 2003), but unlike various earlier VLSM studies in which left- and right-hemisphere damage data was treated jointly (Lo et al., 2010; Cheng et al., 2014; Meyer et al., 2016), this analysis was conducted separately for LHD and RHD patients. The decision to separate the analyses was made under the assumption that differences between the dominant left and the non-dominant right cerebral hemispheres in the functional neuroanatomy of motor control (Tretriluxana et al., 2009; Mani et al., 2013), and in patterns of motor recovery (Zemke et al., 2003; Wu et al., 2015), may be reflected also in a different impact for lesion topography on patients’ capacity to execute SA and FE movements. Indeed, the analysis revealed marked differences between LHD and RHD patient groups in the impact of the lesion pattern on SA and FE expression. This finding is of special interest as it cannot be explained by group differences in baseline parameters. The two hemispheric groups did not differ in a series of demographic data (gender, age, and motor dominance distribution), lesion data (type and total hemispheric volume), time of clinical examination after stroke onset, and HUL impairment level (as reflected in the FM total score, B&B score, and the FM-sensation score). Moreover, the groups’ scores of SA and FE movement capacity did not differ (Table 1), and the two groups showed a similar proportion of patients being affected in all the regions defined by the AAL atlas (Tzourio-Mazoyer et al., 2002; Solomon et al., 2007) and the White-Matter atlas (Mori et al., 2008). The lesion overlay maps of LHD and RHD patient groups (Figure 1) demonstrate a typical stroke lesion pattern with dominant middle-cerebral artery territory damage in both groups.

Despite the above similarities between the groups in baseline parameters, patients with left hemiparesis (LHD) differed significantly from patients with right hemiparesis (RHD) in the impact of lesion topography on the capacity to execute SA and FE movements. In the LHD group, both SA and FE movements were affected mainly by damage to the CST in its passage through the corona radiata. In contrast, SA and FE movements were affected in the RHD group by CST damage both in the corona radiata and the PLIC, as well as by damage to intra-hemispheric association fibers, the insula, and the putamen. As can be seen in Tables s 2, 3, both SA and FE were sensitive, in RHD patients, to damage in a much larger array of brain structures, including many more “significant” voxels, compared to LHD patients. Also, the capacity to execute SA and FE movements correlated negatively with the total hemispheric volume loss in RHD but not in LHD. Given the similarity between the groups in baseline demographic, clinical, and lesion parameters, we interpret the salient difference in the impact of lesion topography on SA and FE expression in LHD and RHD as a reflection of a fundamental difference in structure-function relationships.

We propose that the scarcity of “significant” voxels (i.e., brain voxels in which the existence of damage is shown by VLSM to exert a significant impact on the examined behavior) in the LHD group relative to the RHD group, stems from the fact that in the dominant left hemisphere (most patients in both groups were right-handed), the processing of sensory-motor data is carried out by a more extensive and densely connected network (Guye et al., 2003), where damage to one component is more easily substituted by other network components. Earlier studies have shown that the primary motor cortex, descending corticospinal pathways, somatosensory association, and premotor cortices of the dominant and non-dominant hemispheres differ anatomically and functionally (Serrien et al., 2006). Such differences are reflected in the deeper central sulcus in the dominant hemisphere (Amunts et al., 1996), the more potent intracortical circuits in the primary motor cortex of the dominant hemisphere (Hammond et al., 2004), the more extensive connectivity of the left dominant M1 with other parts of the brain (Guye et al., 2003), the higher excitability of the corticospinal system on the dominant left hemisphere (De Gennaro et al., 2004), and the relationship between the lateralization of the motor network and the quality of performance of different motor tasks (Barber et al., 2012). Recently, we reported on asymmetrical lesion effects in LHD and RHD on overall HUL functioning (Fugl-Meyer and box-and-blocks test scores), which were found both in the sub-acute and the chronic phases following a stroke. As in the current study, the asymmetry reflected a relative paucity of “significant” voxels in the LHD group (Frenkel-Toledo et al., 2019). It should be noted, however, that the current cohort includes subjects who participated also in our aforementioned study, a fact that could contribute to pattern similarity. Our proposed interpretation of the differences between LHD and RHD in the relationship between lesion topography and motor performance relates to hemispheric dominance, thus applying to right-handed patients (forming 93% and 91% of the LHD and RHD cohorts, respectively). With a larger group of left-handed stroke patients, it will be possible to assess whether the above differences are maintained or not among left-handers.

In both left and right hemisphere strokes, damage to the CST affected the capacity to execute SA and FE movements. This finding was expected given the CST role as the main pathway for mediation of cerebral control on the motor neurons of the cervical spinal cord that activate upper limb muscles (Palmer and Ashby, 1992; Lindenberg et al., 2010; Lo et al., 2010; Puig et al., 2011). Earlier studies (e.g., Lo et al., 2010) found that the brain region where damage affects HUL function in the most severe and protracted manner is the area of convergence of the corona radiata into the CST before it enters into the internal capsule. In a recent study, we have found a strong impact on CST damage (in either the corona radiata or the PLIC) on HUL function, following both LHD and RHD. The detrimental effect of CST damage was noted both in the sub-acute and in the chronic phases (Frenkel-Toledo et al., 2019). Algorithms that predict the HUL functional outcome also emphasize the importance of CST integrity (Stinear et al., 2012, 2017b). For example, in the PREP algorithm, patients with a “SAFE score” (i.e., Medical Research Council grades for SA plus FE) higher than 8/10 within 72 h of stroke onset made a “complete” recovery of HUL function within 12 weeks. Patients with a “SAFE score” lower than 8/10, in whom propagation of impulses in the CST could be demonstrated by elicitation of MEPs had a “notable” functional recovery. In patients without elicitable MEPs, diffusion-weighted imaging was required to distinguish patients with “limited” recovery from those with no recovery at all. Thus, incorporation of neurophysiological and neuroimaging techniques to assess the functional integrity of the CST in the first days after onset enables an accurate prediction of HUL function at 3 months after stroke (Stinear et al., 2012).

Careful inspection of cluster coordinates in Tables s 2, 3 reveals location differences when the “significant” voxels for SA and FE within a given structure are compared. To assess further the extent of voxel sharing between SA and FE, “conjunction” analyses were performed (Figure 3, Tables s 4, 5). These analyses differentiated between voxels in which damage affected SA only, FE only, or SA plus FE. The conjunction analyses pointed to a larger proportion of shared voxels in the RHD group (in this group most of the “significant” voxels, within all the involved structures, were shared by both SA and FE). In the LHD group, the number of “significant” voxels was much lower and the proportion of voxels that were found significant to either SA or FE but not to both movements was higher compared to the RHD group. In the LHD group, voxels in which damage affected specifically either SA or FE were found in the superior part of the corona radiata, i.e., near the primary motor cortex, where somatotopic organization separates the representations of proximal and distal upper-limb movements (Snell, 2010). The greater proportion in the LHD group of voxels in which the impact of damage was specific to either SA or FE may point to greater differentiation in the sensory-motor cortex of the dominant hemisphere. Motor dominance in humans (Amunts et al., 1996) and primates (Hopkins and Cantalupo, 2004; Margiotoudi et al., 2019) is attributed to hemispheric asymmetries in motor cortex architecture. On the other hand, the finding, especially following RHD, of voxel sharing for SA and FE (i.e., voxels in which damage affected significantly the capacity of patients to execute both SA and FE movements), reflects the importance of brain structures contributing to these movements in a less specific manner, i.e., both to proximal and distal upper limb movements. Previous research in subacute post-stroke patients found a similar reduction of the active range of motion in proximal and distal segments of the HUL (Beebe and Lang, 2008), while other studies (Colebatch and Gandevia, 1989) reported on greater strength deficits in the more distal HUL muscle groups. Such discordant findings may reflect variant involvement in the stroke process of voxels which contribute to a given movement in a specific manner vs. voxels which contribute to movements in a non-specific manner. Alternatively, such discordant findings may point to the variant impact of damage to homologous voxels in the two hemispheres, as shown in the current study for LHD and RHD patient groups.

It should be noted that the high positive predictive power of SA and FE movement execution is based on the assessment done a few days after stroke onset, whereas the current analyses were conducted in the chronic stage when structure and function in the brain are expected to maintain a more stable relationship. As explained in the introduction section, transient physiological malfunction in “penumbra” regions and not necessarily permanent structural damage, could affect patients’ capacity to execute SA and FE in the acute stage. Thus, theoretically, stroke patients may be unable to execute SA and FE movements in the acute stage even if none of the “significant” voxels disclosed in the current study was damaged permanently. This can explain the fact that early ability to execute SA and FE movements has a strong positive predictive power but a much weaker negative predictive power [for example, Winters et al. (2016) reported that 45% of the patients who could not show voluntary FE about a week after stroke onset, achieved later (at 6 months) a score equal or higher than 10 points in the Action Research Arm Test (ARAT), indicating at least partial HUL functionality]. Given that our lesion data were derived from follow-up brain scans and analyzed against behavioral information obtained months and years after the stroke, it points to structures whose integrity is necessary for the proper execution of SA and FE irrespective of the recovery type (resolution of reversible physiological dysfunction or long-term network re-organization) and magnitude of recovery. Based on the current findings we may assume those stroke patients who could execute SA and FE movements shortly after stroke onset, as well as those who could not execute it initially but regained later HUL dexterity, did not have a major involvement in the location of the “significant” voxel clusters found in the current study.

### Limitations

Several limitations of the study should be acknowledged. First, to avoid spurious results in the VLSM analysis, only voxels damaged in at least 10 subjects were tested. Given the number of subjects in the current cohort (44 LHD, 33 RHD), this threshold precluded an assessment of the impact of damage to relevant brain voxels of the sensory-motor and adjacent cortical regions where the prevalence of damage was lower than this threshold. Also, the majority of the current cohort had strokes located within the MCA territory. This limited the possibility of identifying “significant” voxel clusters related to strokes in other vascular territories, including posterior-circulation brainstem strokes. It is assumed that these limitations lead to type-2 (false negative) errors.

Second, as most subjects had mild to moderate HUL motor impairment at the time of testing (FM scores of 45.0 ± 18.6 and 43.3 ± 24.4 in LHD and RHD groups, respectively; SA and FE median score = 2, for both), patients with more severe hemiparesis were under-represented in this study.

Third, the VLSM results for FE in the LHD group are based on a lenient criterion, as they did not survive the permutation correction for multiple comparisons. These results are likely to reflect a trend that may become significant with larger numbers of subjects, but it may also represent a type-1 (false positive) error.

Fourth, measuring the capacity to execute SA and FE movements using the Medical Research Council (MRC) method (i.e., on a 5-point ordinal scale, James, 2007), as done by Stinear et al. (2012, 2017b), is probably more sensitive to mild improvement compared to the 3-point ordinal scale of the FM used in the current study, and may affect the possibility of comparing the results of studies using these two methods for outcome measurement.

Fifth, data collection in this study was conducted when the participants were in the chronic stage, mostly being tested in their homes. This precluded control of residual aphasia among LHD patients and residual spatial neglect among RHD patients. Both residual aphasia and residual neglect could probably affect the results. For example, the fact that a much larger array of brain structures affected SA and FE movements in the RHD group may be related to residual neglect in part of the RHD cohort. Spatial neglect is known to imply poor functional outcomes for stroke survivors (Katz et al., 1999), emerging from dysfunction within large-scale networks involved in attention, motor, and multimodal sensory processing (Corbetta, 2014).

### Implications

Our findings indicate that the capacity to execute SA and FE movements following stroke is affected differently by lesion topography in LHD and RHD patients. In both groups, these movements are sensitive to damage in brain voxels within the CST. However, in the RHD group, patients’ capacity to execute the movements is affected also by damage to a large array of cortical and subcortical regions. This finding expands previous data pointing to differences between the dominant left and the non-dominant right cerebral hemispheres in the functional neuroanatomy of motor control (Tretriluxana et al., 2009; Mani et al., 2013) and patterns of motor recovery (Zemke et al., 2003; Wu et al., 2015). Our finding that damage to shared and not only to distinct brain voxels affects both SA and FE movement capacity is likely to relate to the quite similar sensitivity and specificity values of these two movements when used as prognostic determinants of overall HUL function (Snickars et al., 2017). Prognostication of HUL function may benefit from adding lesion information to clinical and physiological measures in use, provided that lesion boundaries are already clearly visible in the brain scan, enabling accurate delineation of the area of structural brain damage.

### Summary and Conclusions

The current study sheds new light on the functional neuroanatomy of SA and FE movements. Our findings point to marked differences between left and right hemispheric damage: in the former, SA and FE are affected in the chronic stage mainly by CST damage, while in the latter, the capacity to execute SA and FE movements are affected in addition to CST damage, also by damage to white matter association tracts, the insula, and the putamen. In both groups, voxel clusters are found where damage affects SA and also FE, along with voxels where damage affects only one of the two movements. Voxel specificity is higher in LHD compared to RHD. In LHD, voxels specificity is much higher for FE compared to SA. We propose that the above differences between LHD and RHD stem from physiological differences related to hemispheric motor dominance.

## Data Availability Statement

The raw data supporting the conclusions of this article will be made available by the authors, without undue reservations.

## Ethics Statement

The study was reviewed and approved by the Ethics Review Board of the Loewenstein Hospital (approval number LOE-004-14). The patients/participants provided their written informed consent to participate in this study.

## Author Contributions

SF-T was involved in planning and conducting the experiments as well as data analysis, interpretation, and drafting of the manuscript. SO-G was involved in conducting the experiments as well as data analysis, interpretation of data, and revising of the manuscript. NS was involved in subject medical screening, lesion delineation, planning the experiment, interpretation, and revising of the manuscript. All authors read and approved the final manuscript.

## Conflict of Interest

The authors declare that the research was conducted in the absence of any commercial or financial relationships that could be construed as a potential conflict of interest.
