# Supplementary material for: Lesion Topography Impact on Shoulder Abduction and Finger Extension Following Left and Right Hemispheric Stroke
Source: Front Hum Neurosci. 2020 Jul 17;14:282. doi: 10.3389/fnhum.2020.00282 (PMC7379861; doi:10.3389/fnhum.2020.00282)
Supplement: Supplementary file 1 [file Table_1.DOCX]

**Table S1:**

Individual demographic and clinical data.

| Subject number | Gender | Age (at examination) | Dominance | Lesion side  and  Territory | Lesion type | Lesion volume (cc) | Interval between stroke and CT (days) | TAO (months) | FM SUM | B&B | FM sensation | SA | FE |
| --- | --- | --- | --- | --- | --- | --- | --- | --- | --- | --- | --- | --- | --- |
| 1000 | M | 73 | R | L-MCA | I | 111.61 | 0 | 33.25 | 42 | 3 | 12 | 0 | 1 |
| 1001 | F | 73 | R | L-MCA | I | 34.34 | 30 | 24.33 | 64 | 55 | 12 | 2 | 2 |
| 1002 | M | 58 | R | L-MCA | H | 20.73 | 49 | 42.43 | 46 | 21 | 1 | 0 | 2 |
| 1003 | M | 49 | R | L-MCA | I | 15.20 | 40 | 41.25 | 60 | 67 | 12 | 2 | 2 |
| 1004 | M | 59 | R | L-MCA | I | 35.60 | 7 | 48.03 | 41 | 26 | 12 | 1 | 1 |
| 1005 | F | 43 | R | L-MCA | H | 40.57 | 61 | 52.20 | 58 | 45 | 9 | 2 | 2 |
| 1007 | M | 64 | R | L-MCA | I | 1.42 | 95 | 41.70 | 31 | 18 | 12 | 1 | 1 |
| 1008 | M | 69 | R | L-MCA | H | 75.28 | 44 | 59.77 | 54 | 43 | 10 | 1 | 2 |
| 1009 | F | 45 | R | L-MCA | H | 45.54 | 120 | 36.36 | 29 | 1 | 6 | 0 | 0 |
| 1010 | F | 67 | R | L-MCA | I | 74.31 | 45 | 51.41 | 49 | 25 | 12 | 1 | 2 |
| 1011 | M | 71 | R | L-MCA | H | 69.21 | 24 | 29.90 | 5 | 0 | 12 | 0 | 0 |
| 1012 | M | 69 | R | L-MCA | I | 5.59 | 78 | 57.80 | 65 | 51 | 12 | 2 | 2 |
| 1038 | M | 62 | R | L-BA | H | 0.45 | 38 | 36.59 | 46 | 46 | 10 | 2 | 1 |
| 1041 | F | 60 | R | L-MCA | I | 2.01 | 34 | 16.07 | 58 | NT | NT | 2 | 2 |
| 1042 | M | 68 | R | L-MCA | H | 9.83 | 43 | 32.33 | 16 | 0 | 11 | 0 | 0 |
| 1043 | M | 79 | R | L-MCA | I | 18.54 | 23 | 21.38 | 62 | 42 | 12 | 2 | 2 |
| 1045 | M | 69 | R | L-MCA | I | 9.57 | 1 | 21.93 | 62 | 45 | 12 | 2 | 2 |
| 1048 | M | 27 | R | L-MCA | I | 5.21 | 51 | 15.41 | 60 | 52 | 10 | 2 | 2 |
| 1049 | M | 61 | R | L-MCA | I | 4.02 | 0 | 17.11 | 3 | 0 | 12 | 0 | 0 |
| 1051 | M | 51 | R | L-MCA | H | 7.13 | 73 | 20.23 | 26 | 0 | 2 | 1 | 0 |
| 1055 | M | 63 | R | L-MCA | I | 6.14 | 3 | 36.57 | 23 | 6 | 8 | 0 | 0 |
| 1057 | F | 57 | R | L-MCA-PCA WS | I | 22.91 | 0 | 33.26 | 63 | 63 | 12 | 2 | 2 |
| 1058 | F | 58 | R | L-MCA | I | 7.27 | 77 | 13.08 | 48 | NT | NT | 1 | 2 |
| 1059 | M | 47 | R | L-MCA | I | 5.23 | 1 | 14.46 | 32 | NT | 0 | 2 | 1 |
| 1061 | M | 47 | R | L-MCA | H | 11.61 | 49 | 13.54 | 42 | NT | 0 | 2 | 1 |
| 1063 | M | 67 | R | L-MCA | H | 29.65 | 43 | 14.52 | 56 | 32 | 10 | 2 | 2 |
| 1065 | M | 68 | R | L-MCA | H | 13.86 | 56 | 11.97 | 22 | 0 | 6 | 0 | 1 |
| 1069 | M | 65 | R | L-MCA | H | 4.67 | 67 | 26.56 | 49 | 18 | 4 | 2 | 2 |
| 1070 | M | 62 | R | L-MCA | I | 41.37 | 567 | 25.64 | 62 | 49 | 12 | 2 | 2 |
| 1071 | M | 61 | R | L-MCA | I | 8.30 | 0 | 23.64 | 66 | 56 | 12 | 2 | 2 |
| 1073 | F | 52 | R | L-MCA | H | 37.49 | 98 | 12.53 | 13 | 0 | 12 | 0 | 0 |
| 1074 | M | 66 | R | L-MCA | I | 57.77 | 60 | 36.49 | 45 | 12 | 4 | 1 | 2 |
| 1075 | M | 65 | L | L-MCA | I | 22.87 | 45 | 34.75 | 66 | 62 | 12 | 2 | 2 |
| 1076 | M | 55 | R | L-MCA | I | 1.26 | 0 | 18.49 | 54 | 51 | 12 | 2 | 1 |
| 1077 | F | 56 | L | L-MCA | I | 0.97 | 22 | 19.41 | 62 | 63 | 12 | 2 | 2 |
| 1079 | F | 52 | R | L-MCA | I | 3.35 | 4 | 14.26 | 34 | NT | 12 | 0 | 2 |
| 1080 | F | 60 | R | L-MCA | I | 5.42 | 52 | 14.16 | 56 | NT | 7 | 2 | 2 |
| 1081 | M | 51 | R | L-MCA | I | 21.19 | 49 | 26.17 | 19 | 0 | 0 | 0 | 0 |
| 1083 | M | 40 | R | L-MCA | H | 21.71 | 38 | 12.60 | 7 | 0 | 7 | 0 | 1 |
| 1085 | M | 70 | R | L-MCA | H | 38.71 | 42 | 46.20 | 65 | 58 | 3 | 2 | 2 |
| 1086 | M | 59 | R | L-PCA | H | 24.10 | 59 | 17.77 | 58 | 36 | 5 | 2 | 2 |
| 1087 | M | 47 | R | L-BA | I | 0.36 | 3 | 14.87 | 60 | NT | NT | 2 | 2 |
| 1088 | M | 78 | ambi | L-MCA-PCA | I/H | 182.27 | 42 | 15.10 | 43 | NT | NT | 1 | 2 |
| 1089 | F | 63 | R | L-MCA | I | 5.23 | 27 | 14.47 | 57 | NT | NT | 2 | 2 |
| 2000 | F | 54 | R | R-MCA | I | 12.43 | 3 | 54.00 | 49 | 26 | 8 | 2 | 1 |
| 2001 | M | 68 | R | R-MCA | H | 23.39 | 43 | 55.93 | 20 | 5 | 10 | 0 | 1 |
| 2002 | F | 67 | R | R-BA | I | 1.18 | 33 | 51.80 | 50 | 35 | 12 | 1 | 1 |
| 2003 | M | 59 | R | R-MCA | H | 1.19 | 56 | 32.30 | 58 | 29 | 9 | 2 | 2 |
| 2004 | M | 27 | R | R-MCA | I | 0.98 | 14 | 45.28 | 65 | 62 | 12 | 2 | 1 |
| 2005 | M | 73 | R | R-MCA | I/H | 93.16 | 40 | 68.46 | 4 | 0 | 2 | 0 | 0 |
| 2006 | M | 68 | R | R-MCA | I | 92.41 | 71 | 31.02 | 66 | 54 | 12 | 2 | 2 |
| 2036 | F | 73 | ambi | R-MCA | I | 70.55 | 5 | 33.77 | 63 | 42 | 8 | 2 | 2 |
| 2041 | M | 56 | R | R-MCA | H | 4.86 | 43 | 26.43 | 54 | 55 | 10 | 2 | 2 |
| 2043 | M | 68 | R | R-MCA | I | 26.16 | 25 | 24.00 | 66 | 37 | 12 | 2 | 2 |
| 2044 | M | 68 | R | R-MCA | I | 0.28 | 34 | 24.00 | 60 | 31 | 12 | 2 | 2 |
| 2046 | M | 63 | R | R-MCA | H | 13.52 | 42 | 16.00 | 63 | 52 | 12 | 2 | 2 |
| 2047 | F | 61 | R | R-MCA | H | 17.86 | 42 | 13.54 | 64 | 46 | 10 | 2 | 2 |
| 2051 | M | 67 | R | R-MCA | I | 1.89 | 0 | 24.69 | 61 | 47 | 12 | 2 | 2 |
| 2052 | M | 69 | R | R-MCA | I | 44.79 | 3 | 23.97 | 61 | 24 | 10 | 2 | 2 |
| 2053 | M | 61 | R | R-MCA | I | 0.99 | 5 | 22.85 | 66 | 56 | 12 | 2 | 2 |
| 2055 | F | 52 | R | R-MCA | I | 11.87 | 58 | 13.11 | 57 | NT | NT | 2 | 2 |
| 2057 | F | 73 | R | R-MCA | I | 1.36 | 24 | 14.00 | 58 | NT | NT | 2 | 2 |
| 2058 | M | 58 | R | R-MCA | H | 24.57 | 40 | 19.41 | 7 | 0 | 6 | 0 | 0 |
| 2060 | M | 59 | R | R-MCA | I | 0.78 | 0 | 13.77 | 62 | 54 | 12 | 2 | 2 |
| 2065 | M | 67 | R | R-MCA | I | 4.70 | 1 | 25.70 | 65 | 53 | 12 | 2 | 2 |
| 2067 | F | 69 | R | R-MCA | I | 12.41 | 1 | 24.98 | 6 | 0 | 5 | 0 | 0 |
| 2070 | M | 57 | L | R-MCA | I | 92.09 | 5 | 24.82 | 8 | 0 | 6 | 0 | 0 |
| 2071 | M | 71 | R | R-MCA | I | 186.89 | 20 | 23.61 | 6 | 0 | 5 | 0 | 0 |
| 2072 | M | 68 | R | R-MCA | I>H | 80.85 | 30 | 23.18 | 6 | 0 | 9 | 0 | 0 |
| 2074 | M | 40 | L | R-MCA | H | 69.57 | 49 | 39.02 | 7 | 0 | 2 | 0 | 0 |
| 2075 | M | 65 | R | R-MCA | I | 1.45 | 35 | 45.93 | 53 | 26 | 4 | 2 | 2 |
| 2076 | M | 67 | R | R-MCA | H | 30.92 | 43 | 39.87 | 4 | 0 | 2 | 0 | 0 |
| 2078 | M | 68 | R | R-MCA | I | 2.44 | 0 | 28.95 | 62 | 40 | 12 | 2 | 2 |
| 2079 | M | 70 | R | R-MCA | I | 20.92 | 25 | 31.84 | 59 | 53 | 12 | 2 | 2 |
| 2080 | F | 67 | R | R-MCA | H | 25.76 | 55 | 14.23 | 21 | 0 | 3 | 0 | 1 |
| 2081 | M | 68 | R | R-MCA | H | 70.44 | 45 | 35.37 | 25 | 0 | 5 | 0 | 0 |
| 2082 | M | 63 | R | R-MCA | I | 1.65 | 30 | 13.67 | 54 | NT | NT | 2 | 2 |

M - male; F - female; MCA - middle cerebral artery; PCA - posterior cerebral artery; BA - basilar artery; WS – water shadow; H - hemorrhagic stroke; I - ischemic stroke; I/H - ischemic with hemorrhagic transformation; TAO - time after stroke onset (months); FM - Fugl-Meyer (see Methods); B&B - Box and Blocks; SA - shoulder abduction, FE - finger extension; NT – not tested.
