# Supplementary figures and images for: Lesion Topography Impact on Shoulder Abduction and Finger Extension Following Left and Right Hemispheric Stroke
Source: Front Hum Neurosci. 2020 Jul 17;14:282. doi: 10.3389/fnhum.2020.00282 (PMC7379861; doi:10.3389/fnhum.2020.00282)

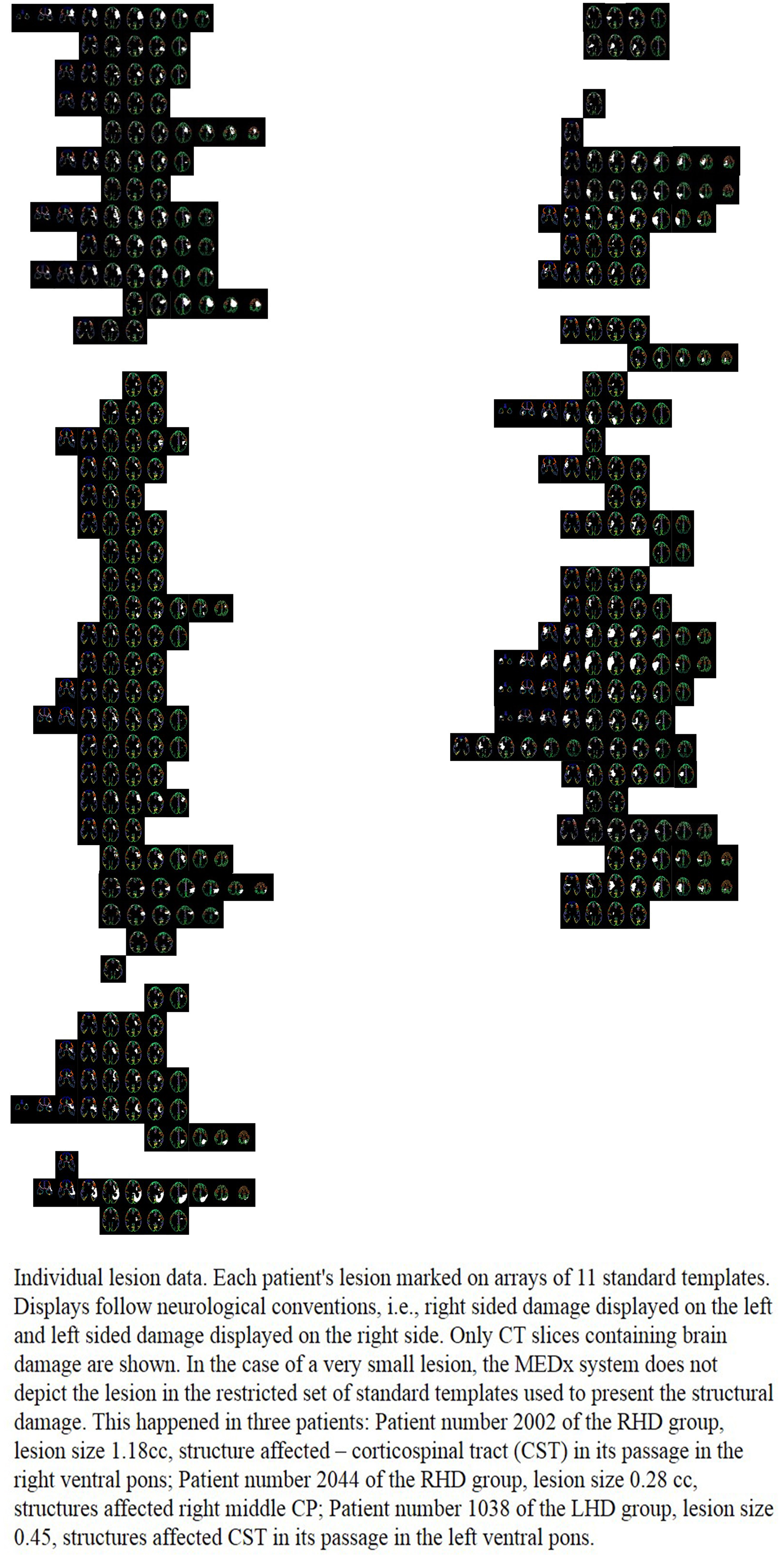

Supplement: Supplementary file 2 [file Image_1.jpg]
